# Pan-Cancer Analysis of Voltage-Dependent Anion Channel (VDAC1) as a Cancer Therapeutic Target or Diagnostic Biomarker

**DOI:** 10.1155/2022/5946110

**Published:** 2022-07-31

**Authors:** Zhitong Wang, Yinchu Cheng, Zaiwei Song, Rongsheng Zhao

**Affiliations:** Department of Pharmacy, Peking University Third Hospital; Institute for Drug Evaluation, Peking University Health Science Center; Therapeutic Drug Monitoring and Clinical Toxicology Center, Peking University, Beijing 100191, China

## Abstract

The voltage-dependent anion channel 1 (VDAC1), a pore protein located in the outer mitochondrial membrane, has been confirmed to be related to cancer in cell or animal evidence. However, there is no available pan-cancer analysis of VDAC1. Herein, we investigated the potential roles of VDAC1 in tumorigenesis and progression based on the Cancer Genome Atlas (TCGA), Gene Expression Omnibus (GEO), and Clinical Proteomic Tumor Analysis Consortium (CPTAC) datasets. The expression of VDAC1 increased in most cancers, and the upregulation of VDAC1 distinctly correlated with the poor prognosis in patients, including breast invasive carcinoma, cervical squamous cell carcinoma, pancreatic adenocarcinoma, lung adenocarcinoma, and skin cutaneous melanoma. We also found VDAC1 S104 phosphorylation raised in various cancers, such as breast cancer, colon cancer, and lung adenocarcinoma. Moreover, the expression of VDAC1 was related to the estimated infiltration value of cancer-associated fibroblasts in bladder urothelial carcinoma, colon adenocarcinoma, kidney renal papillary cell carcinoma, and testicular germ cell tumors. At last, we showed that VDAC1-related oxidative phosphorylation and metabolic regulation may partially explain its association with tumorigenesis and progression. Taken together, this pan-cancer analysis provides relatively comprehensive information on the potential value of VDAC1 as a prognostic biomarker and therapeutic target.

## 1. Introduction

Due to the lengthening of life expectancy and the complexity of tumorigenesis, the morbidity of malignant tumors is increasing rapidly worldwide, with low therapeutic success worldwide [1]. Pan-cancer research conducts an integrated analysis of diverse types of cancer to identify the common characteristics and heterogeneity of various cancer cell lineages through different omics such as the genome, transcriptome, and proteome. Recently, pan-cancer analysis has been utilized to determine certain functional genes and molecular pathways, which helps to understand human cancer comprehensively. As available research tools, some public databases, including Gene Expression Omnibus (GEO), the Cancer Genome Atlas (TCGA), and the Clinical Proteomic Tumor Analysis Consortium (CPTAC), provide high-throughput gene expression and functional genomics data sets in different tumor types [2, 3].

The voltage-dependent anion channel (VDAC) is a major pore protein located at the outer membrane of the mitochondrion, which is described in eukaryotes of all species [4]. In mammals, three isoforms of VDACs have been identified, namely, VDAC1, VDAC2, and VDAC3. Among them, VDAC1 is the most abundant and best-characterized one. As a channel for metabolites transport across the outer mitochondrial membrane, VDAC1 is one of the main regulators of mitochondrial metabolism [5]. Previous studies state that cancer cell metabolism is characterized by partial inhibition of mitochondrial metabolism and enhanced aerobic glycolysis, called the Warburg effect. Therefore, changes in VDAC expression or conformational state are related to cancer metabolism regulation [6]. Indeed, VDAC1 was overexpressed in cervical cancer, nonsmall cell lung cancer, and other malignant tumors, suggesting that VDAC1 plays a vital role in high-energy demand tumor cells [7, 8].

In addition, VDAC1 is also a critical protein in mitochondria-mediated apoptosis, involving the release of apoptogenic factors and interaction with antiapoptotic proteins. By forming PTP, hetero-oligomerization with proapoptotic proteins (Bax), or homo-oligomerization, VDAC1 participates in forming channels large enough in the outer mitochondrial membrane to release apoptotic proteins, such as Cyto c and apoptosis-inducing factor. VDAC also acts as an anchor point for antiapoptotic proteins, such as HK, Bcl2, and Bcl-xL [9]. It has been proved that VDAC1-based peptides in the cytoplasm eliminated the ability of cells to evade apoptotic pathways because of the specific binding of free VDAC1-peptides with antiapoptotic proteins that interferes with their activity [10]. One characteristic distinguishing cancer cells from normal tissue cells is the imbalance of apoptotic and antiapoptotic proteins. Cancer cells typically possess high antiapoptotic proteins, which may require more VDAC1 in the outer mitochondrion membrane to provide more anchor points. Because of the functions of VDAC1 in cell metabolism and apoptosis, it is considered to be involved in the progression of cancer and is a potential anticancer therapeutic target [11]. However, there has been no systematic pan-cancer focus on VDAC1 to clarify its potential clinical value in treatment and prognosis.

This study analyzed VDAC1 in pan-cancer using the TCGA program and GEO database for the first time. Furthermore, the potential characteristic information and molecular mechanisms of VDAC1 in the onset or prognosis of cancers were explored, such as gene expression, survival analysis, alteration, phosphorylation, immune infiltration, and related cellular signal pathways.

## 2. Materials and Methods

### 2.1. *VDAC1* Expression Analysis

We obtained the genome location information of the VDAC1 gene based on the UCSC (http://genome.ucsc.edu/) on human GRCh38/hg38 (Dec. 2013) assembly [12]. Additionally, we performed an amino acid sequence alignment of VDAC1 in different species, which was applied by the “ClustalW” function in the MEGA7 program [13]. Furthermore, the phylogenetic tree of VDAC1 in different species was obtained by using the constraint-based multiple alignment online tool of the NCBI (https://www.ncbi.nlm.nih.gov/tools/cobalt/). The Human Protein Atlas (HPA) online dataset (https://www.proteinatlas.org/humanproteome/pathology) was used to obtain the VDAC1 expression data across different tissues and cells under physiological conditions. The expression level of VDAC1 protein in plasma samples was estimated in the HPA database by proteomics determination based on mass spectrometry. Herein, “Low specificity” was defined by “normalized expression ≥1 in at least one tissue/region/cell type but not elevated in any tissue/region/cell type.”

In the TCGA project, “VDAC1” was input in the “Gene_DE” module of tumor immune estimation resource, version 2 (TIMER2) web (http://timer.cistrome.org/) to obtain the expression changes of VDAC1 in different tumor tissues and corresponding normal tissues. Due to the limited control data of some normal tissues in the TCGA database, such as cervical and endocervical cancers (CESC), lymphoid neoplasm diffuse large B-cell lymphoma (DLBC), acute myeloid leukemia (LAML), pancreatic adenocarcinoma (PAAD), skin cutaneous melanoma (SKCM), and testicular germ cell tumors (TGCT), we use the “Expression analysis-Box Plots” module of the GEPIA2 web tool (http://gepia2.cancer-pku.cn/#analysis) to combine TCGA and genotype-tissue expression (GTEx) datasets for analysis. In the “Expression analysis-Box Plots” module of GEPIA2, “*P*-value cutoff” = 0.01, ^“^log2FC (fold change) cutoff^”^ = 1, and “Match TCGA normal and GTEx data” was selected to obtain box plots of VDAC1 expression in tumor or normal tissues. Moreover, violin plots of the VDAC1 expression of all TCGA tumor types at different pathological stages were obtained from the “Pathological Stage Plot” module of GEPIA2.

Next, the “Total-protein” module in the UALCAN tool (http://ualcan.path.uab.edu/index.html) was used to analyze the VDAC1 protein expression in the CPTAC database [14]. Additionally, the expression level of VDAC1 phosphoprotein analysis (with phosphorylation at the S104, S102) in primary tumor tissues was also performed via selecting the “phosphoprotein” module and entering “VDAC1.” A total of six tumor datasets available in CPTAC were selected, including breast cancer, colon cancer, clear cell renal cell carcinoma (RCC), lung adenocarcinoma, ovarian cancer, and uterine corpus endometrial carcinoma (UCEC).

### 2.2. VDAC1 Survival-Associated Analysis

The overall survival (OS) and disease-free survival (DFS) data of VDAC1 based on TCGA were obtained by using the “Survival Map” function in the “Survival Analysis” module of GEPIA2 [15]. The expression thresholds were set to 50% of the cutoff-high and cutoff-low values as segmentation of high-expression and low-expression cohorts [16]. In addition, the survival plots were obtained under the “Survival Analysis” module in GEPIA2 by inputting VDAC1. The hypothesis testing method selected for survival analysis was the log-rank test. Additionally, the Kaplan-Meier plotter (http://kmplot.com/analysis/) was used to perform a variety of survival analyses, including OS, relapse-free survival (RFS), first progression (FP), progress-free survival (PFS), and postprogression survival (PPS) in breast, gastric, liver, lung, and ovarian cancers. We obtained the Kaplan-Meier survival plots and the data of *P* value, the hazard ratio (HR), and 95% confidence intervals by selecting “auto select best cutoff” to slit patients.

### 2.3. Genetic Alteration of VDAC1

The genetic alteration analysis of VDAC1 was performed by the “TCGA Pan-Cancer Atlas Studies” module of the cBioPortal web tool (https://www.cbioportal.org/) [17]. The alteration frequency and mutation types in all TCGA tumors were supplied under the “Cancer Types Summary” module. In addition, we used the three-dimensional (3D) structure of VDAC1 to display the mutated site information as the schematic diagram via the “Mutations” module. The “comparison/survival” module obtained the overall, disease-specific, disease-free, and progression-free survival with or without VDAC1 genetic alteration in selected cancer types were obtained by the “comparison/survival” module. For the correlation analysis between VDAC1 expression and tumor mutational burden (TMB) or microsatellite instability (MSI), the tumor RNA-seq data of different TCGA tumors were obtained from the Genomic Data Commons data portal (https://portal.gdc.cancer.gov/) [18]. The statistical analysis was performed by R software v4.0.3. The rank-sum test detected two data sets, and *P* < 0.05 was statistically significant.

### 2.4. Immune Infiltration Analysis of VDAC1

The association between *VDAC1* and immune infiltrates in the TCGA database was investigated by the “Immune-Gene” module of the TIMER2 web [19]. After selecting CD8^+^ T-cells or cancer-associated fibroblasts as the cell type for immune infiltration, the EPIC, MCPCOUNTER, and XCELL algorithms were used to analyze immune infiltration. A heat map in tumors visualized the data obtained through different algorithms, and the data of certain tumors with the same changing trend was further displayed as a scatter plot. The Spearman's rank correlation test was performed to obtain partial correlation (cor) values and the *P* values.

### 2.5. VDAC1-Related Gene Enrichment Analysis

The STRING website (https://string-db.org/) was used to determine VDAC1-binding proteins [20]. First, we inputted the protein name “VDAC1” and selected the organism as “Homo sapiens.” The main parameters were set in sequence, including the meaning of network edges (“evidence”), active interaction sources (“experiments”), the minimum required interaction score (“Low confidence (0.150)”), and the max number of interactors to show (“no more than 50 interactors” in the first shell). Next, the VDAC1-related genes of all TCGA tumors were obtained via the “Similar Gene Detection” module in GEPIA2. In addition, a pairwise gene Pearson correlation analysis of VDAC1 and selected genes was performed via the “correlation analysis” module of GEPIA2. The correlation coefficient (*R*) and the *P* value were represented as log2 TPM in the dot plot. Moreover, the heat map data of the selected genes was obtained through the “Gene_Corr” module in TIMER2, and the *P* value and partial correlation (cor) were calculated through the Spearman's rank correlation test.

An interactive Venn diagram was used to perform an intersection analysis of the VDAC1-binding proteins and related genes [21]. Based on the above two parts of data, a functional annotation chart was obtained by uploading a gene list and selecting identifier (“OFFICIAL_GENE_SYMBOL”) and species (“Homo sapiens”) in the DAVID database (https://david.ncifcrf.gov/) [18]. Then, we used R packages of “tidyr” and “ggplot2” in R language software [R-3.6.3] (https://www.r-project.org/) to visualize the enriched pathways. The “clusterProfiler” R package was applied to conduct Gene Ontology (GO) enrichment analysis, and cnetplots were used to visualize biological process (BP), cellular component (CC), and molecular function (MF). *P* < 0.05 was considered to be statistically significant.

## 3. Results

### 3.1. Gene Expression Features of VDAC1

**Figure 1:**
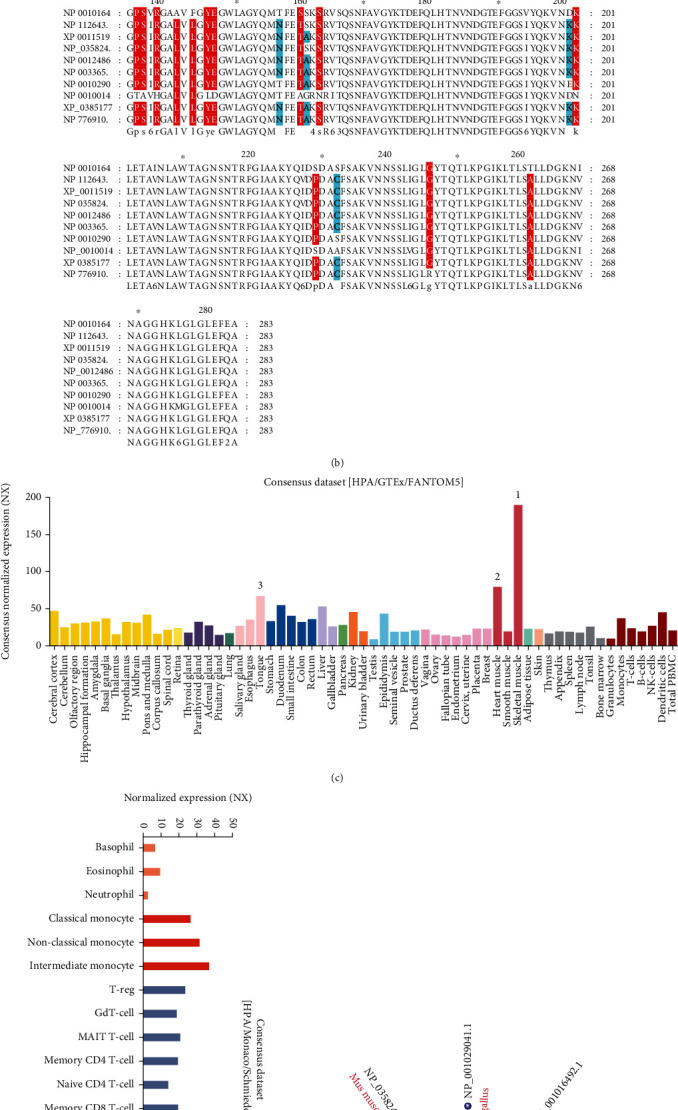
Structural characteristics, phylogenetic tree, and expression level of VDAC1. (a) The location of VDAC1 in the human genome. (b) Amino acid sequence alignment of VDAC1 among different species. (c) The expression of the VDAC1 among different tissues was obtained from the consensus database of HPA, GTEx, and FANTOM5. (d) The expression of the VDAC1 in blood cells was observed from the consensus database of HPA, Monaco, and Schmiedel. (e) VDAC1 phylogenetic tree among different species was obtained by a constraint-based multiple alignment tool in NCBI.

In this study, human VDAC1 gene expression (mRNA: NM_003374.3 protein: NP_003365.1, Figure 1(a)) in normal and oncogenesis was explored. The results provided by the amino acid sequence alignment indicated that the protein structure of VDAC1 in different species was conserved (Figure 1(b)). Then, the expression pattern of VDAC1 was analyzed in other normal tissues. The consensus dataset of the HPA, GTEx, and function annotation of the mammalian genome 5 (FANTOM5) showed VDAC1 was the highest expression in the skeletal muscle, followed by the heart muscle (Figure 1(c)), suggesting that the abundance of VDAC1 expression in muscle tissues with more mitochondrial content was higher than other tissues. However, VDAC1 expression can be detected in the tissues involved in the dataset, indicating its low tissue specificity. Furthermore, the expression analysis of VDAC1 in different blood cells also demonstrated low blood cell type specificity by analyzing the consensus dataset of the HPA/Monaco/Schmiedel datasets (Figure 1(d)). In addition, the phylogenetic tree of VDAC1 protein showed the evolutionary relationship in species (Figure 1(e)).

### 3.2. Gene Expression Data of VDAC1 in Pan-Cancer

**Figure 2:**
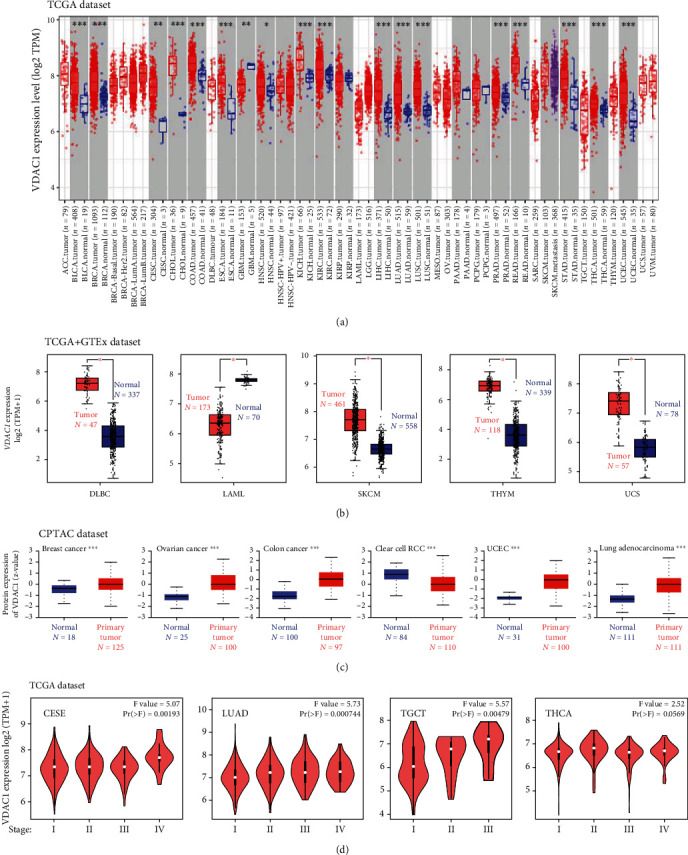
Expression level of VDAC1 among different tumors. (a) VDAC1 expression among different cancer types was analyzed by TIMER2. (b) For the cancer types of CESC, DLBC, LAML, PAAD, SKCM, THYM, and UCS in the TCGA, the box plot data were observed through the joint application analysis of GTEx and TCGA database. (c) The expression level of VDAC1 protein was analyzed in normal or primary tumor tissues of breast cancer, ovarian cancer, colon cancer, clear cell RCC, UCEC, and lung adenocarcinoma based on the CPTAC dataset. (d) The main pathological stages in VDAC1 expression among different cancers was obtained based on TCGA dataset. ^∗^*p* < 0.05; ^∗∗^*p* < 0.01; ^∗∗∗^*p* < 0.001.

The TIMER2 approach was applied to analyze VDAC1 expression in various cancer types of TCGA. As shown in Figure 2(a), the expression of VDAC1 was upregulated in most tumor tissues, including bladder urothelial carcinoma (BLCA), breast invasive carcinoma (BRCA), cholangiocarcinoma (CHOL), colon adenocarcinoma (COAD), esophageal carcinoma (ESCA), kidney chromophobe (KICH), kidney renal clear cell carcinoma (KIRC), liver hepatocellular carcinoma (LIHC), lung adenocarcinoma (LUAD), lung squamous cell carcinoma (LUSC), prostate adenocarcinoma (PRAD), rectum adenocarcinoma (READ), stomach adenocarcinoma (STAD), thyroid carcinoma (THCA), UCEC (*P* < 0.001), CESC, glioblastoma multiforme (GBM) (*P* < 0.01), and head and neck squamous cell carcinoma (HNSC) (*P* < 0.05) compared with corresponding normal tissues. Due to the missing part of control data in certain cancer types of TCGA, including adrenocortical carcinoma (ACC), DLBC, LAML, brain lower-grade glioma (LGG), ovarian serous cystadenocarcinoma (OV), sarcoma (SARC), SKCM, TGCT, thymoma (THYM), and uterine carcinosarcoma (UCS), the expression analysis of VDAC1 was performed between the tumor tissues and normal tissues by combining the TCGA and GTEx datasets. As shown in Figure 2(b), the expression levels of VDAC1 among the tumor tissues, such as CESC, DLBC, LAML, PAAD, SKCM, THYM, and UCS, were significantly different from those in the corresponding control tissues (*P* < 0.05). However, no significant difference was obtained for other tumors, including ACC, GBM, OV, LGG, or TGCT (Supplementary Figure 1a).

The results of the Clinical Proteomic Tumor Analysis Consortium (CPTAC) database shown in Figure 2(c) indicated that the expression of VDAC1 protein increased in primary tumors, including breast cancer, ovarian cancer, colon cancer, UCEC, and LUAD, and reduced in clear cell RCC, compared with normal tissues (*P* < 0.001).  Figure 2(d) demonstrated the statistical correlation between VDAC1 expression and the pathological stages of cancers, such as CESE, LUAD, and TGCT, through the pathological stage plot of GEPIA2 (*P* < 0.05). In addition, the data of different pathological stages in THCA also indicated a specific correlation with the expression of VDAC1 (*P* = 0.0569). No significant difference was obtained for other tumors shown in Supplementary Figure 1b.

### 3.3. VDAC1 Survival-Associated Analysis

**Figure 3:**
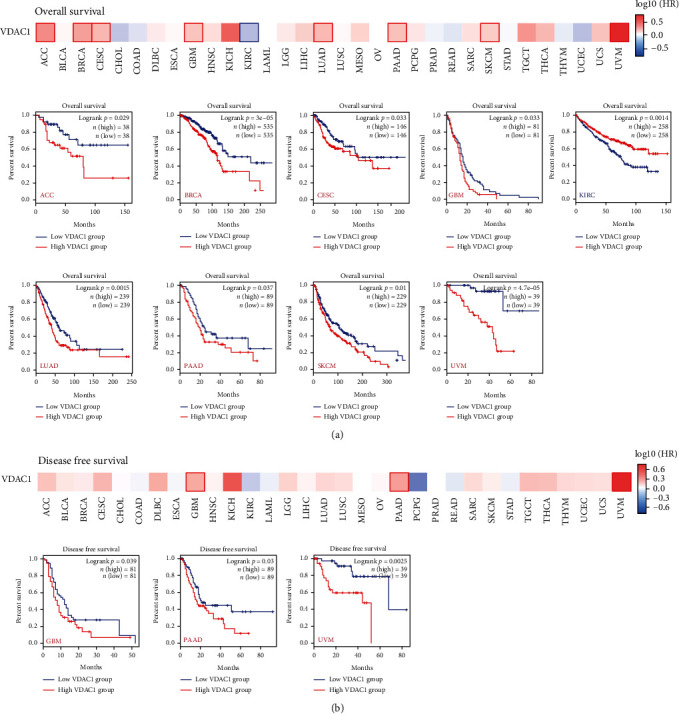
Correlation between VDAC1 expression and survival tumor prognosis in TCGA. The survival plots and Kaplan-Meier curves showed the statistical correlation between overall survival (a) or disease-free survival analysis (b) VDAC1 expression among different cancers using the GEPIA2 tool.

Next, the cancer cases of the TCGA and GEO datasets were divided into high or low VDAC1 expression groups, and the correlation between VDAC1 expression and the prognosis of different tumors was investigated. As shown in Figure 3(a), high expression of VDAC1 was related to the poor overall survival of ACC, BRCA, CESC, GBM, LUAD, PAAD, SKCM, and UVM cases, and low VDAC1 expression was associated with a poor overall survival prognosis of KIRC cases based on the TCGA project (*P* < 0.05). Additionally, the results of DFS analysis demonstrated a correlation of high-expressed VDAC1 with poor prognosis of GBM, PAAD, and UVM (*P* < 0.05) in the TCGA cases (Figure 3(b)).

The survival analysis of the Kaplan-Meier plotter implied a correlation between high VDAC1 expression and poor prognosis of different cancers, e.g., breast cancer: OS (*P* = 0.013), RFS (*P* = 3.8*e* − 10), and PPS (*P* = 0.0011) as shown in Supplementary Figure 2a; ovarian cancer: PFS (*P* = 0.0033) and PPS (*P* = 0.046) as shown in Supplementary Figure 2b; lung cancer: OS (*P* = 7.1*e* − 5) and PPS (*P* = 0.0001) as shown in Supplementary Figure 2c; or liver cancer: OS (*P* = 0.019) as shown in Supplementary Figure 2e. In contrast, Supplementary Figure 2d indicated that the positive correlation between VDAC1 expression and OS (*P* = 5.9*e* − 10), FP (*P* = 5.5*e* − 9), and PPS (*P* = 6.1*e* − 13) in gastric cancer. Additionally, a series of subgroup analyses were performed by distinct clinical factors and demonstrated the detailed correlation between expression levels of VDAC1 and selected cancer types (Supplementary Table 1–5). These results indicated that VDAC1 expression is differentially related to the prognosis with distinct tumor types, suggesting that VDAC1 could be used as a potential biomarker for the prognostic diagnosis of certain cancer types.

### 3.4. Genetic Alteration of VDAC1

**Figure 4:**
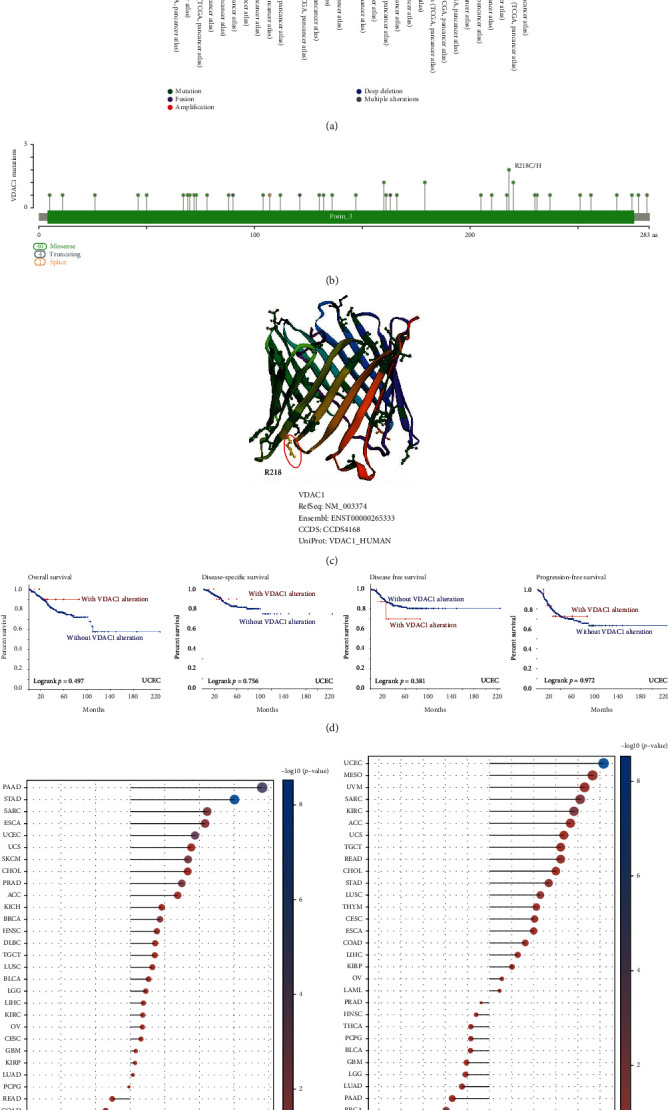
Mutation features of VDAC1 in TCGA tumor types. The alteration frequency (a) and VDAC1 mutation sites (b) of different mutation types among TCGA tumors were obtained by the cBioPortal tool. The mutation site with the highest alteration frequency (R218) was shown in the 3D structure of VDAC1 (c). In addition, the survival curves of overall, disease-specific, disease-free, and progression-free associated with VDAC1 mutation status in UCEC were obtained by using the cBioPortal tool (d). Moreover, the potential correlation between VDAC1 expression and tumor mutational burden (TMB) (e) or microsatellite instability (MSI) (f) was analyzed based on the TCGA.

The genetic alteration of VDAC1 was observed in tumor samples in TCGA cohorts.  Figure 4(a) indicated the highest alteration frequency of VDAC1 (>5%) with amplification as the primary type in kidney renal clear cell carcinoma cases. The mutation as the primary type in the uterine corpus endometrial carcinoma showed an alteration frequency of ~2%. As shown in Figure 4(b), the sites, types, and case numbers of the VDAC1 genetic alteration were further presented. The missense of VDAC1 was found as the primary type of alteration, and alteration of R218C/H at the 218 site of VDAC1 protein was detected in papillary thyroid cancer, uterine endometrioid carcinoma, and mucinous adenocarcinoma of the colon and rectum. The R218 site was presented in the three dimensions structure model of VDAC1 protein (Figure 4(c)). Moreover, Figure 4(d) indicated that UCEC cases with altered VDAC1 had no significant changes in overall and disease-specific survival, disease-free, and progression-free survival, compared with patients without VDAC1 alteration. These data suggest that the association between VDAC1 gene alteration and the survival prognosis of different cancer types requires further exploration in a larger number of cancer case samples.

TMB was considered to be an independent predictor of immunotherapy effectiveness in different cancer types and also MSI served as a specific indicator to predict the efficacy of immune checkpoint inhibitors in diverse cancers [22, 23]. Next, we analyzed the correlation between TMB/MSI and VDAC1 expression in all tumor types of TCGA. As shown in Figure 4(e), a positive correlation was observed between VDAC1 expression and TMB for BRCA (*P* = 0.0068), ESCA (*P* = 0.0077), PAAD (*P* = 8.3*e* − 07), PRAD (*P* = 0.001), SARC (*P* = 0.0007), SKCM (*P* = 0.0003), STAD (*P* = 4.5*e* − 09), and UCEC (*P* = 2.1*e* − 05); and a negative correlation was found for THCA (*P* = 0.009), THYM (*P* = 0.006), and UVM (*P* = 0.017). In addition, VDAC1 expression was positively correlated with MSI in KIRC (*P* = 0.00045), LUSC (*P* = 0.010), MESO (*P* = 0.035), READ (*P* = 0.048), STAD (*P* = 0.0095), and UCEC (*P* = 1.19*e* − 09), but was negatively correlated with BRCA (*P* = 0.0016) (Figure 4(f)). These results suggest the correlation between VDAC1 and tumor immunotherapy, but more in-depth research is worthwhile to verify.

### 3.5. Protein Phosphorylation of VDAC1

**Figure 5:**
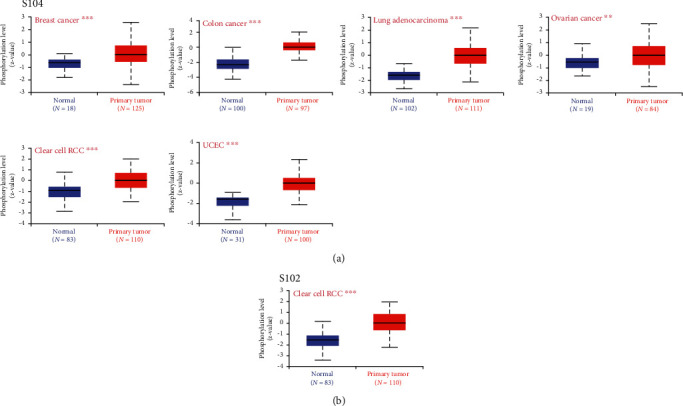
Phosphorylation levels of VDAC1 protein in different tumors. According to the CPTAC dataset, the UALCAN tool provides boxplots (median, 25-75% range) of VDAC1 phosphorylation levels of S104 (a) and S102 (b) in certain cancer types such as breast cancer, colon cancer, lung adenocarcinoma, ovarian cancer, clear cell RCC, and UCEC.

The phosphorylation levels of VDAC1 between primary tumor tissues and normal tissues were compared based on the CPTAC. This database analyzed five tumors, including breast cancer, lung adenocarcinoma, clear cell renal cell carcinoma, uterine corpus endometrial carcinoma, and ovarian cancer. As shown in Figure 5(a), the VDAC1 phosphorylation level at S104 increased in primary tumor tissues of breast cancer (*P* = 1.05*e* − 05), colon cancer (*P* = 8.6*e* − 36), lung adenocarcinoma (*P* = 1.2*e* − 22), ovarian cancer (*P* = 1.07*e* − 02), clear cell renal cell carcinoma (*P* = 1.7*e* − 13), and uterine corpus endometrial carcinoma (*P* = 6.4*e* − 10), compared with normal tissues. Additionally, an increase in the phosphorylation level of S102 was observed in clear cell renal cell carcinoma (Figure 5(b), *P* = 2.3*e* − 17). However, no data was retrieved using UALCAN network resources for other tumors, including breast cancer, lung adenocarcinoma, uterine corpus endometrial carcinoma, and ovarian cancer. The possible reason is that there is no relevant data has been uploaded to the CPTAC database before. Furthermore, the PhosphoNET database was used to detect phosphorylation levels of VDAC1 in CPTAC. The results showed that based on experiments, VDAC1 phosphorylation at S104 or S102 was supported by a paper, respectively [24, 25] (Supplementary Table 6). These results deserve further molecular assays to explore the correlation between the S104 or S102 phosphorylation site of VDAC1 and tumorigenesis.

### 3.6. Immune Infiltration Analysis of VDAC1

**Figure 6:**
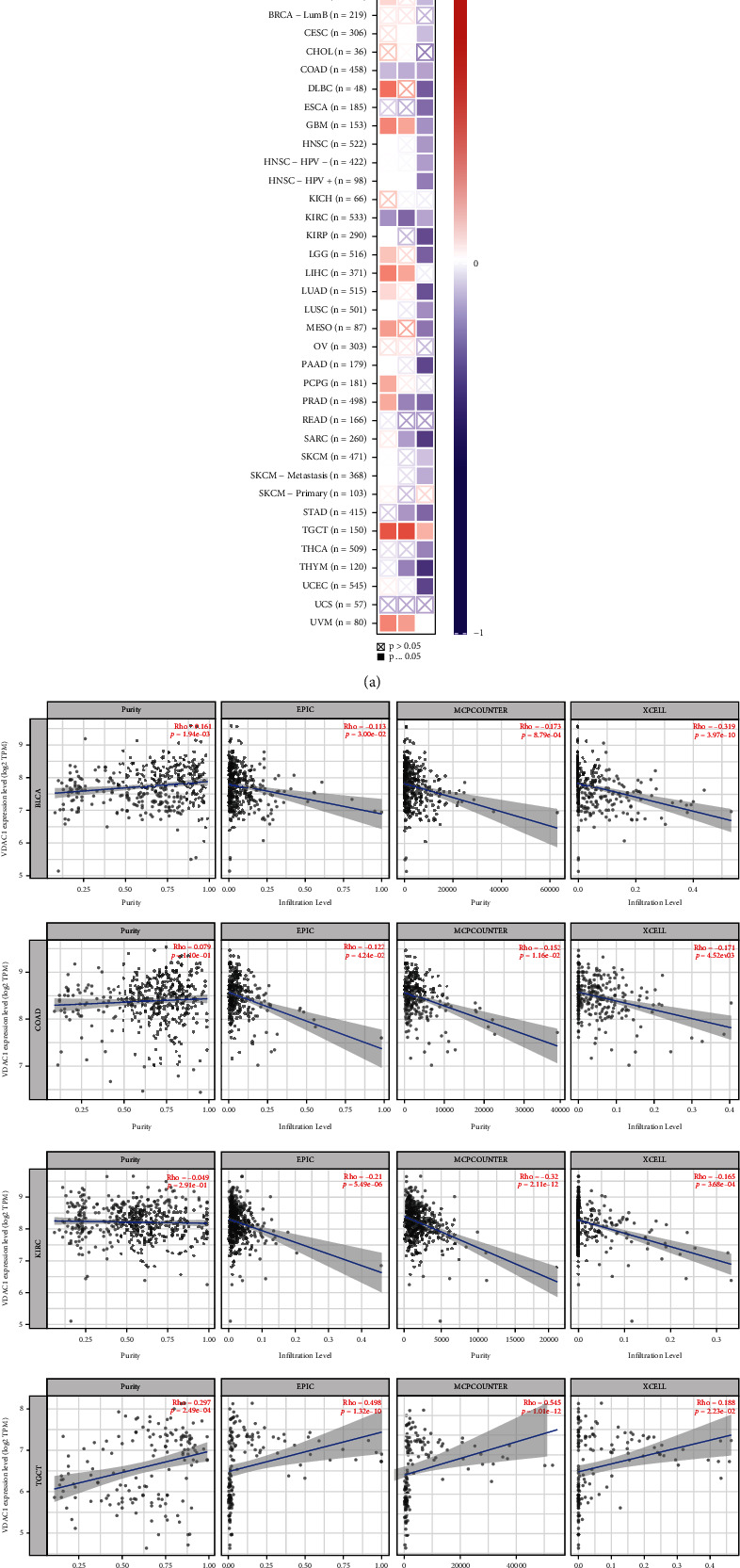
Correlation between VDAC1 expression and the immune infiltration of cancer-associated fibroblasts. The corresponding heat map (a) and scatter plots (b) of the relationship between the expression of VDAC1 and the infiltration level of cancer-associated fibroblasts in different types of TCGA cancer were explored via different algorithms.

As an essential part of the tumor microenvironment, tumor-infiltrating immune cells were closely related to the occurrence, progression, or metastasis of cancer and participated in the regulation of various tumor-infiltrating immune cell functions [26]. As shown in Figure 6, the scatterplot data in different cancer types of TCGA was produced based on specific algorithms, such as EPIC, XCELL, and MCPCOUNTER. The results showed a negative correlation between VDAC1 expression and the estimated infiltration value of cancer-associated fibroblasts in BLCA, COAD, and KIRC, but a positive association with TGCT. Moreover, we observed a negative correlation between VDAC1 expression and CD8^+^ T-cells immune infiltration in PAAD, but a positive correlation with UVM through the majority of algorithms (Supplementary Figures 3a and 3b). As shown in Supplementary Figure 3c, the correlation between VDAC1 expression and different immune cells infiltration in TCGA cancers was analyzed, such as CD4^+^ T-cell, regulatory T-cell, T follicular helper cell, gamma delta T-cell, natural killer T-cell, B cell, neutrophil, monocyte, macrophage, dendritic cell, natural killer cell, mast cell, and eosinophil. In most algorithms, a positive correlation between VDAC1 expression and regulatory T-cell immune infiltration could be observed in pheochromocytoma and paraganglioma (PCPG). In addition, the expression of VDAC1 was positively correlated with the infiltration of macrophages (M0 and M1) in STAD and neutrophils in BRCA-basal. On the contrary, the level of VDAC1 in TGCT was negatively correlated with B cell immune infiltration. These results suggest the role of VDAC1 in the tumor microenvironment and its potential correlation with predictions of immunotherapy for certain tumor types.

### 3.7. Enrichment Analysis of VDAC1

**Figure 7:**
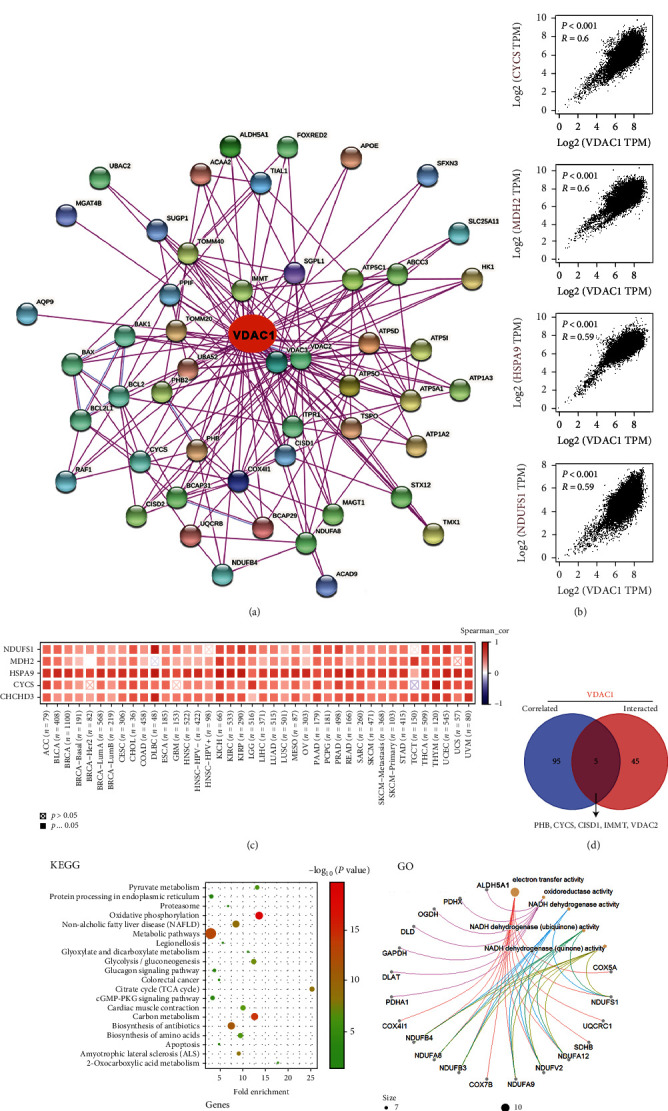
Enrichment analysis of VDAC1-related genes. (a) The experimentally determined VDAC1-binding proteins were obtained by the STRING tool. (b) The VDAC1-related genes in TCGA projects were obtained through the GEPIA2 approach. In addition, the expression correlation analysis between VDAC1 and selected targeting genes, such as CHCHD3, CYCS, HSPA9, MDH2, and NDUFS, was conducted. (c) The corresponding heat map in the majority of cancers of TCGA and GTEx was supplied by TIMER tool. (d) The intersection analysis of the VDAC1-correlated genes and VDAC1-binding proteins was performed. (e) The effects of VDAC1 on tumor pathogenesis ware displayed by KEGG pathway analysis based on the VDAC1-related genes and proteins. (f) Furthermore, the net plot for the molecular function data was explored through GO enrichment analysis.

Moreover, we conducted a signal pathway enrichment analysis based on VDAC1 interacting proteins and VDAC1 expression-related genes to further study the molecular mechanism of the VDAC1 gene in cancer progression.  Figure 7(a) indicated the top 50 VDAC1-binding proteins obtained through the STRING tool and presented as an interaction network. Then, the gene expression profiling interactive analysis, version 2 (GEPIA2) tool, was used to obtain the top 100 genes related to VDAC1 expression in various cancer types of TCGA. As shown in Figure 7(b), the VDAC1 expression was positively correlated with coiled-coil-helix-coiled-coil-helix domain containing 3 (CHCHD3, *R* = 0.6), malate dehydrogenase 2 (MDH2, *R* = 0.6), cytochrome c, somatic (CYCS, *R* = 0.6), heat shock 70 kDa protein 9 (HSPA9, *R* = 0.59), and NADH dehydrogenase (ubiquinone) iron-sulfur protein genes (NDUFS, *R* = 0.59, all *P* < 0.001). The corresponding heat map data also represented a positive correlation between VDAC1 expression and the selected genes in different cancers of TCGA and GTEx (Figure 7(c)). The intersection analysis in the top 100 VDAC1-related genes and 50 VDAC1-binding proteins obtained five common members: *PHB*, *CYCS*, *CISD1*, *IMMT*, and *VDAC2* (Figure 7(d)). Next, we combined the two datasets to conduct Kyoto Encyclopedia of Genes and Genomes (KEGG) and GO enrichment analyses. As shown in Figure 7(e), the KEGG data suggested that “oxidative phosphorylation” and “metabolic pathways” were involved in the role of VDAC1 in the pathogenesis of cancer. Furthermore, the GO enrichment analysis demonstrated that the majority of the selected genes was associated with the pathways or molecular function of oxidative phosphorylation, including electron transfer activity, NADH dehydrogenase (ubiquinone/quinone) activity, cellular respiration, and mitochondrial protein-containing complex (Figure 7(f) and Supplementary Figure 4).

## 4. Discussion

It has been reported that VDAC1 is an essential regulator in the cross-talk of bioenergetics metabolism and apoptosis signal between cytoplasm and mitochondrion [27]. Multiple studies have indicated that VDAC1 is related to tumor progression, and molecules, peptides, and mRNAs targeting VDAC1 are potentially effective in cancer treatment [11]. VDAC has been identified as a highly conserved channel protein [28]. Our study also proved that the protein structure of VDAC1 was conservative among different species, suggesting that the normal physiological function of VDAC1 might involve a similar mechanism. However, whether VDAC1 participates in the pathogenesis of various tumors in clinical cases via some common molecular mechanisms remains unknown. Thus, a pan-cancer study of VDAC1 was performed, including gene expression and alteration, protein phosphorylation, survival prognosis analysis, immune infiltration, or molecular pathway enrichment in different tumor types based on the TCGA, GEO, and CPTAC datasets.

VDAC1 was highly expressed in most tumor tissues and could serve as a potential biomarker for prognostic diagnosis. Previously, Ko et al. systematically analyzed the expression pattern of VDAC1 and indicated that VDAC1 expression was increased in breast, colon, liver, lung, pancreatic, and thyroid cancers. Multiple VDAC1 interacting genes were differentially expressed in tumor tissues [29]. In this study, the increase in VDAC1 expression was also confirmed in most tumor tissues based on the analysis of TCGA and GTEx databases. On this basis, combined with survival analysis, our results indicated a correlation between highly VDAC1 expressed and poor survival prognosis in BRCA, CESC, LUAD, PAAD, and SKCM. Current studies suggested that the possible mechanism of VDAC1 upregulation in cancer was related to providing metabolic advantages to tumor cells. VDAC1 plays a crucial role in regulating mitochondrial energy metabolism; it controls the transport of metabolites across the mitochondrial outer membrane and forms complexes with a variety of metabolism-related proteins, including hexokinase (HK, a key enzyme in glycolysis). It has been well-documented that the Warburg effect in tumor cells leads to enhanced glycolysis and upregulation of HK expression [30]. As the mitochondrial anchoring site of HK, VDAC1 forms a complex with HK, which enables HK to obtain preferential access to mitochondrial ATP and protection from product inhibition, thereby pushing glucose metabolism towards glycolysis [31]. Therefore, it was proposed that the HK-binding capacity of malignant tumor cell mitochondria is associated with an increased number of VDAC binding sites, contributing to maintaining the highly glycolytic phenotype [32]. In normal cells, VDAC1 overexpression increases the level of oligomerization, leading to higher permeability of the mitochondrial outer membrane and mediating the release of apoptogenic factors and caspase activation. It was previously demonstrated that tumor cells upregulated the expression levels of antiapoptotic proteins such as anti-apoptotic members of the Bcl-2 family and HK [33]. These antiapoptotic proteins were proposed to bind to the VDAC1, inhibit its oligomerization, block the release of mitochondrial apoptosis proteins such as cytochrome c, and protect against mitochondria-mediated apoptosis [4]. Therefore, overexpression of VDAC1 did not promote the release of apoptotic factors but increased the energy supply of glycolysis, which was beneficial to tumor cells.

In breast cancer, according to the survival data of the Kaplan-Meier plotter (E-MTAB-365, GSE11121, GSE12093, GSE12267, GSE1456, etc.), our analysis indicated that high expression of VDAC1 was related to poor OS, RFS, and distant metastasis-free survival (DMFS) of cases, especially in a subgroup analysis of “TP53 wild type,” “PR negative,” “HER2 negative,” “grade 3,” “Intrinsic subtype/Luminal A,” “Pietenpol subtype/Basal-like 2,” and “Pietenpol subtype/Mesenchymal stem-like” (Supplementary Figure 2b and Supplementary Table 1). Similarly, it has been reported that VDAC1 is highly expressed in breast cancer tissues, which may predict the poor prognosis of breast cancer cases, especially triple-negative breast cancer [34]. In addition, the results in vitro and mouse models indicated that silencing VDAC1 expression inhibited the growth of different cancer cells, including triple-negative breast cancer, suggesting VDAC1 was involved in the reprogramming of breast cancer cells metabolism, regardless of origin or mutational status [35].

For lung cancer, previous studies have reported the correlation between VDAC1 expression and the initiation and progression of nonsmall cell lung cancer [8]. This study observed a correlation between highly VDAC1 expressed and poor overall survival prognosis of lung adenocarcinoma (*P* = 0.0015) but not lung squamous cell carcinoma by analyzing the LUSC and LUAD datasets in the TCGA project (Figure 2(b)). In addition, a Cox regression survival analysis in the TCGA-LUAD cohort was performed by web OncoLnc (http://www.oncolnc.org/), demonstrating a statistical correlation with VDAC1 (Cox coefficient = 0.328, *P* = 2.60*e* − 05). Furthermore, we performed a group of survival analyses through the Kaplan-Meier plotter approach and observed that high expression of VDAC1 was related to poor OS, FP, and PPS in cases of lung adenocarcinoma (Supplementary Table 2). All these results suggested that VDAC1 might serve as a potential prognosis marker in lung adenocarcinoma cases, consistent with the proteomics results of lung adenocarcinoma tissue [36]. However, the correlation of VDAC1 in different lung cancer types may require a larger sample size to verify.

It has been reported that highly expressed VDAC1 exacerbated the clinical progression of cervical cancer patients [37]. In vitro, the cervical cancer cell line (HeLa) highly expresses VDAC, and VDAC1 silencing has been proved to inhibit cancer cell proliferation [7]. In this study, TCGA-based survival analysis by the GEPIA2 tool suggested that high expression of VDAC1 was linked to poor survival prognosis of CESC (Figure 2(a)). In addition, according to the analysis of the Kaplan-Meier plotter, VDAC1 expression was related to the clinical prognosis of PFS and PPS, but no correlation with OS in cervical cancer (Supplementary Figure 2b). However, no statistical correlation was detected in a Cox regression survival analysis in the TCGA-CESC cohort by web OncoLnc (*P* = 0.083). We speculated that different data processing or unsynchronized survival information updates might impact the results. Therefore, larger sample sizes are required to reveal the role of VDAC1 in the prognosis of cervical cancer patients.

Recent studies have demonstrated that some tumor-related signaling pathways alter the phosphorylation level of VDAC1, leading to cell metabolism rearrangement and affecting apoptosis and cell cycle regulation [38, 39]. Our results showed VDAC1 protein expression and phosphorylation level at the S104 were elevated in breast cancer, colon cancer, lung adenocarcinoma, ovarian cancer, and uterine corpus endometrial carcinoma except for clear cell renal cell carcinoma. Similarly, a previous study reported low VDAC1 expression levels were linked to poor prognosis in kidney renal clear cell carcinoma cases, and the mechanism might involve the hypoxia-cleaved form of VDAC1 induced the resorption of the primary cilium in a Hypoxia-Inducible Factor-1 dependent manner [40]. Further studies have indicated that the hypoxia-induced cleaved form of VDAC1 was due to the loss of the putative phosphorylation site at serine 215 while reducing the interaction with tubulin and microtubules reprogramming the cell to utilize more metabolites, which is beneficial to the cell growing under hypoxic conditions [41]. These hypotheses suggested that VDAC1 expression and phosphorylation might affect the occurrence and progression of tumors by changing the interaction and activity of metabolic molecules. In addition, our results indicate the role of VDAC1 phosphorylation levels at the S104 and S102 in tumorigenesis, and the related molecular mechanisms require to be confirmed by further studies.

This study also provided an analysis of MSI or TMB associated with VDAC1 expression in different TCGA tumor types, providing clues for VDAC1-related tumor immunotherapy. It has been reported that VDAC1, as the mitochondrial anchor site of HK, plays an essential role in maintaining the high glycolysis phenotype of tumor cells [4]. The glycolytic activity was associated with active immune signatures in different cancers, suggesting VDAC1 may participate in tumor immune-related mechanisms through metabolic regulation [42]. In addition, our results indicated that VDAC1 expression correlated with the immune infiltration of cancer-associated fibroblasts, suggesting the potential impact of VDAC1 in regulating the tumor microenvironment. Based on the data of VDAC1-binding proteins and the VDAC1-related genes, the molecular pathway enrichment results demonstrated the potential role of “oxidative phosphorylation” and “metabolic pathways” in tumorigenesis and progression. Similarly, the deletion or mutation of the VDAC1 gene (*POR1*) in yeast has been proved to disturb OXPHOS complex expression, leading to TCA cycle impoverishment [43].

However, even though we comprehensively analyzed information from multiple databases, this study still has limitations. First, this study only used bioinformatics methods to analyze the correlation between VDAC1 expression and prognosis of cancer patients in different databases, and no in vitro or in vivo validation experiments were performed. Future studies of VDAC1 at the cellular and molecular levels will help elucidate its value in cancer diagnosis and therapy. Second, although VDAC1 protein was highly conserved across species, its multiple mutation types and mutation sites were obtained in TCGA cancers, as shown in Figure 4. Due to the small sample size, we were not able to reach a definite conclusion on the association of VDAC1 mutations with prognosis in cancer patients. Third, this study only analyzed the clinical data related to VDAC1 protein expression in different cancer types. The changes in VDAC1 channel conductance and crucial protein interactions in different cancer clinical samples were not involved. Prospective studies focusing on VDAC1 protein function and interaction can be conducted in the future to comprehensively elucidate the potential oncogenic roles of VDAC1 and provide evidentiary support for the development of VDAC1-related small molecule compounds and interfering peptides.

In summary, this pan-cancer analysis clarified the correlation between VDAC1 expression level and the prognosis of different cancer types. Furthermore, by analyzing protein phosphorylation, mutation, and molecular mechanism of VDAC1 in tumor tissues, as well as the correlation of VDAC1 expression with TMB/MSI and immune infiltration, it helps to understand the role of VDAC1 in tumorigenesis and progression and provides evidence to support VDAC1 as a potential prognostic biomarker and therapeutic target.

## Data Availability

The datasets in this study can be obtained from the listed database: TCGA, CPTAC, cBioPortal, UALCAN, GEO, GTEx, HPA, KEGG, and Kaplan-Meier Plotter.

## Supplementary Materials

Supplementary Materials Supplementary Figure 1: VDAC1 expression across different tumors and pathological stages. Supplementary Figure 2: prognosis analysis related to VDAC1 expression through the Kaplan-Meier plotter. Supplementary Figure 3: correlation between VDAC1 expression and immune cells infiltration. Supplementary Figure 4: GO enrichment analysis of VDAC1 related genes in a biological process or cellular component. Supplementary Table 1: subgroup analysis on the correlation of VDAC1 expression and prognosis of breast cancer cases. Supplementary Table 2: subgroup analysis on the correlation of VDAC1 expression and prognosis of lung cancer cases. Supplementary Table 3: subgroup analysis on the correlation of VDAC1 expression and prognosis of ovarian cancer cases. Supplementary Table 4: subgroup analysis on the correlation of VDAC1 expression and prognosis of gastric cancer cases. Supplementary Table 5: subgroup analysis on the correlation of VDAC1 expression and prognosis of liver cancer cases. Supplementary Table 6: analysis of CPTAC-identified phosphorylation sites of VDAC1 via the PhosphoNET database.
