# Supplementary material for: Pan-Cancer Analysis of Voltage-Dependent Anion Channel (VDAC1) as a Cancer Therapeutic Target or Diagnostic Biomarker
Source: Dis Markers. 2022 Jul 31;2022:5946110. doi: 10.1155/2022/5946110 (PMC9357804; doi:10.1155/2022/5946110)
Supplement: Supplementary Materials — Supplementary Figure 1: VDAC1 expression across different tumors and pathological stages. Supplementary Figure 2: prognosis analysis related to VDAC1 expression through the Kaplan-Meier plotter. Supplementary Figure 3: correlation between VDAC1 expression and immune cells infiltration. Supplementary Figure 4: GO enrichment analysis of VDAC1 related genes in a biological process or cellular component. Supplementary Table 1: subgroup analysis on the correlation of VDAC1 expression and prognosis of breast cancer cases. Supplementary Table 2: subgroup analysis on the correlation of VDAC1 expression and prognosis of lung cancer cases. Supplementary Table 3: subgroup analysis on the correlation of VDAC1 expression and prognosis of ovarian cancer cases. Supplementary Table 4: subgroup analysis on the correlation of VDAC1 expression and prognosis of gastric cancer cases. Supplementary Table 5: subgroup analysis on the correlation of VDAC1 expression and prognosis of liver cancer cases. Supplementary Table 6: analysis of CPTAC-identified phosphorylation sites of VDAC1 via the PhosphoNET database. [file 5946110.f1.docx]

Supplementary Material

# Supplementary Figure


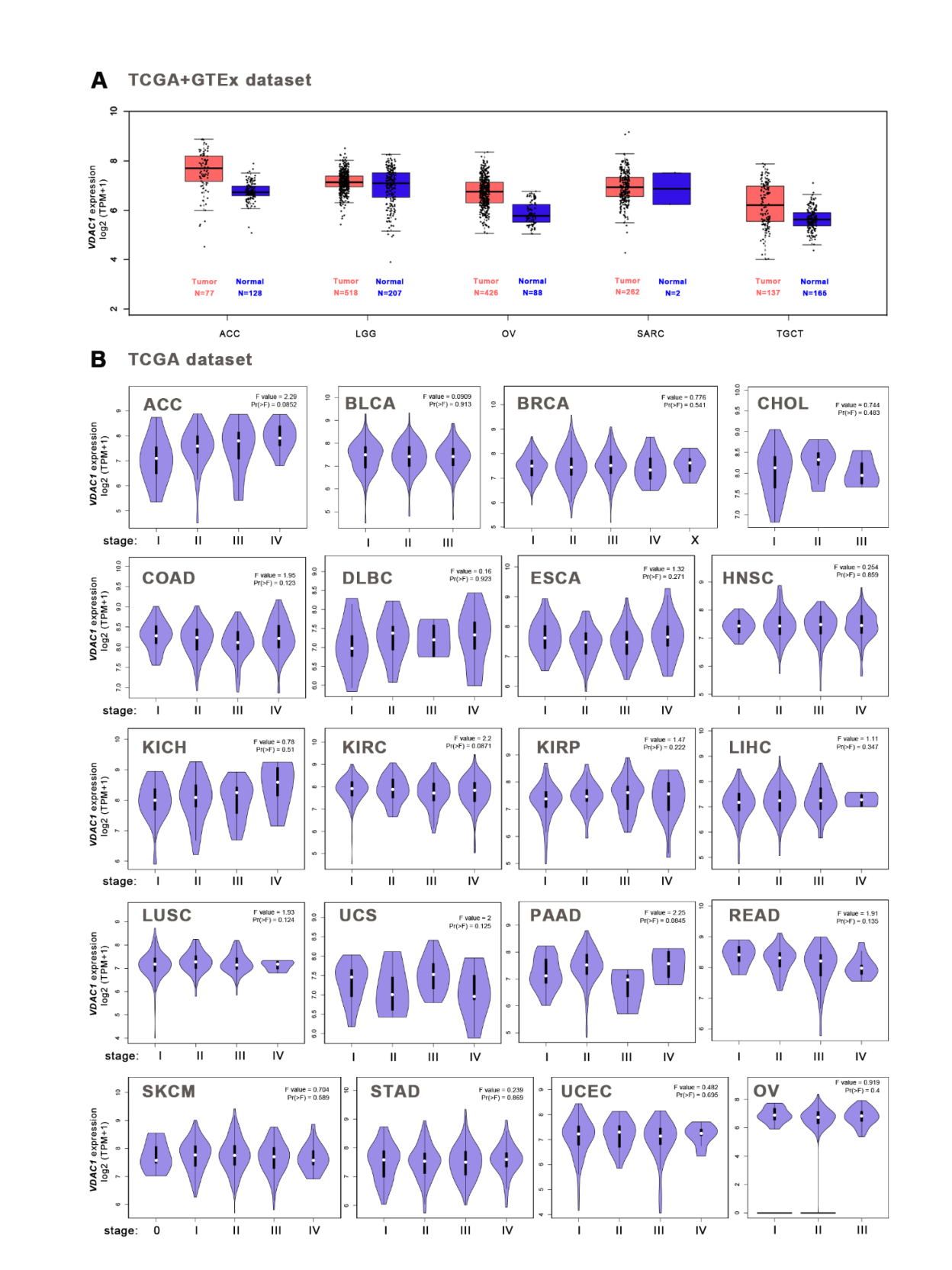


**Supplementary Figure 1**. VDAC1 expression across different tumors and pathological stages. **(A)** The boxplots of VDAC1 expression in the cancers of ACC, GBM, OV, LGG and TGCT were obtained through the combined GTEx and TCGA database analysis. **(B)** The expression levels of the VDAC1 in the main pathological stages of ACC, BLCA, BRCA, CHOL, COAD, DLBC, ESCA, HNSC, KICH, KIRC, KIRP, LIHC, LUSC, UCS, PAAD, READ, SKCM, STAD, UCEC and OV were obtained based on TCGA dataset.


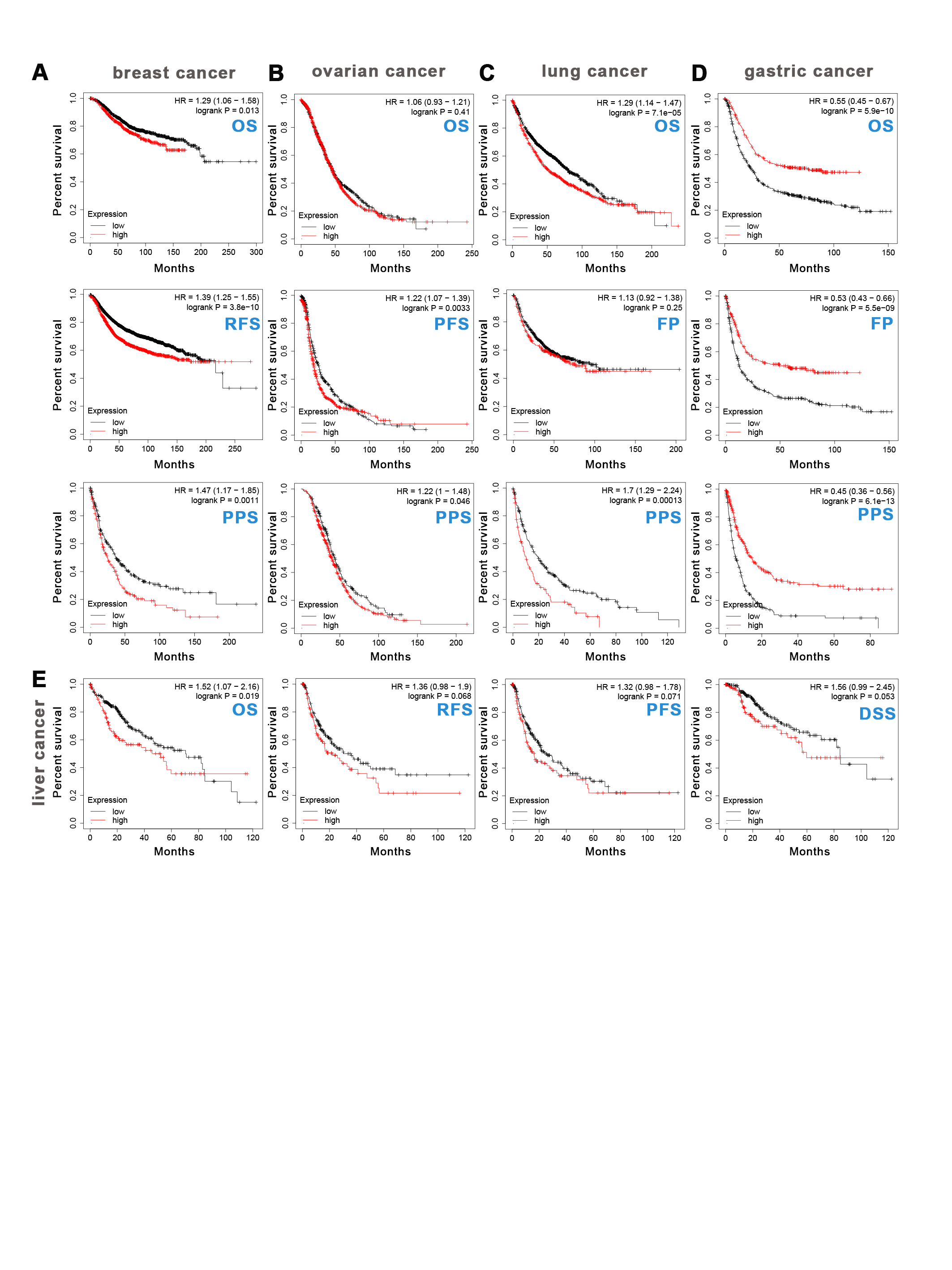


**Supplementary Figure 2.** Prognosis analysis related to VDAC1 expression through the Kaplan-Meier plotter. The survival prognosis analysis associated with VDAC1 expression, such as OS (overall survival), RFS (relapse-free survival), PFS (progress free survival), DMFS (distant metastasis-free survival), PPS (post-progression survival), FP (first progression), and DSS (disease-specific survival) was obtained by the Kaplan-Meier plotter in various cancer types, including breast cancer, gastric cancer, liver cancer, lung cancer and ovarian cancer based on GEO datasets **(A-E).**


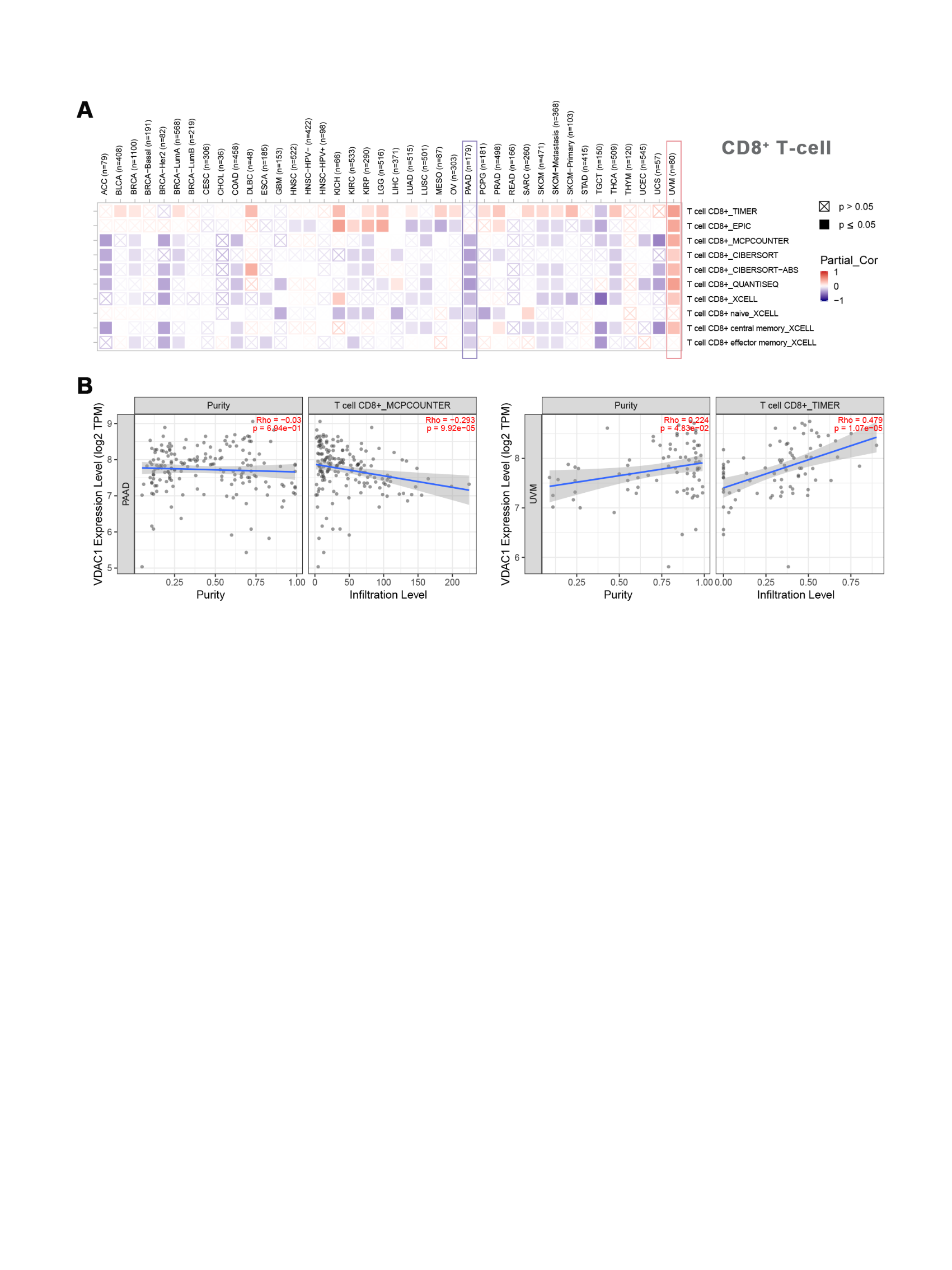


**
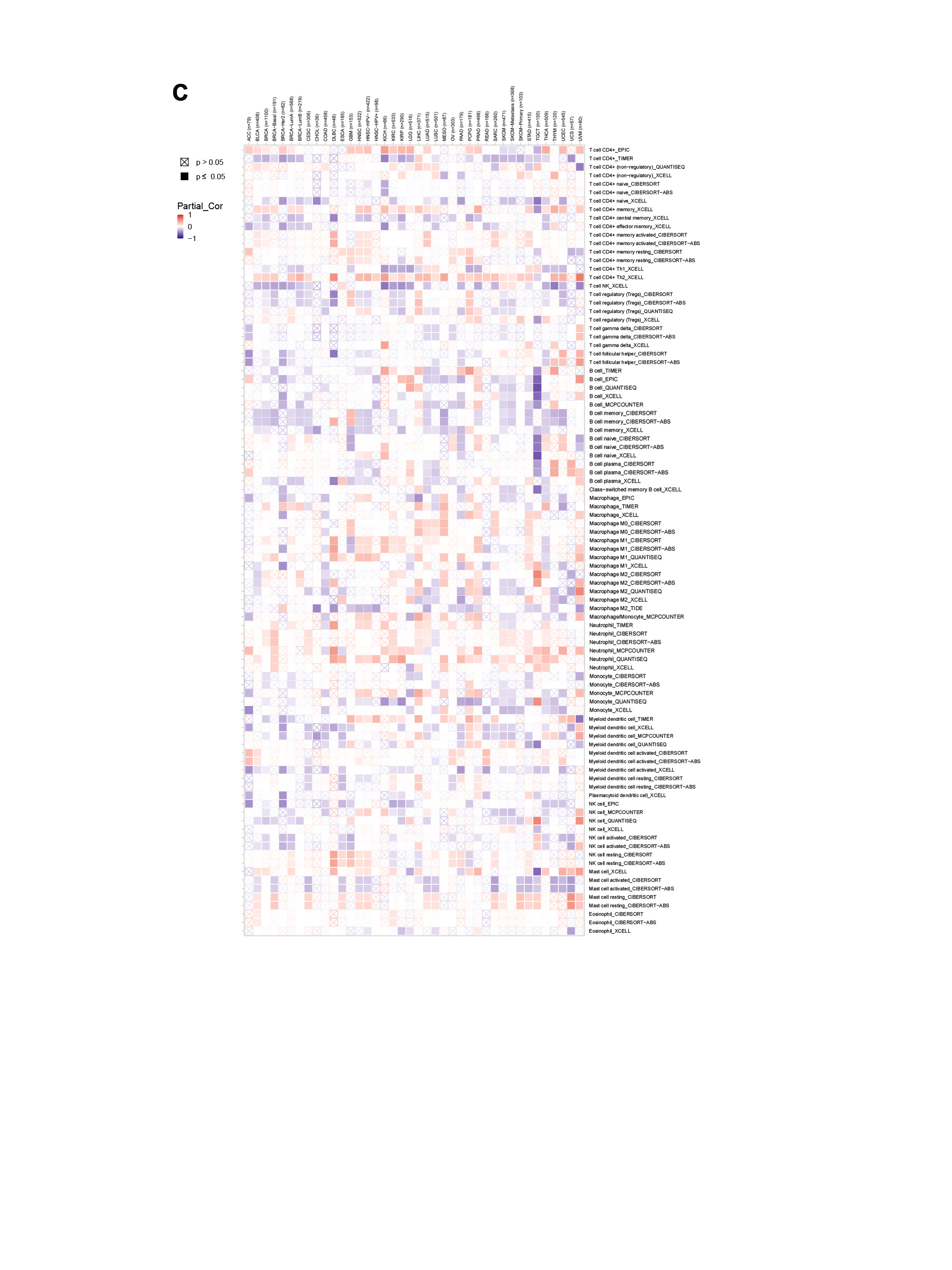
**

**Supplementary Figure 3.** Correlation between VDAC1 expression and immune cells infiltration. The heat map **(A)** and scatter plots **(B)** of the correlation between VDAC1 expression and CD8^+^ T-cell infiltration in TCGA cancers were obtained through various algorithms. **(C)** The heat map of the correlation of VDAC1 expression with different immune cells infiltration in TCGA cancers, such as CD4^+^ T-cell, regulatory T-cell, T follicular helper cell, gamma delta T-cell, natural killer T-cell, B cell, neutrophil, monocyte, macrophage, dendritic cell, natural killer cell, mast cell, and eosinophil.


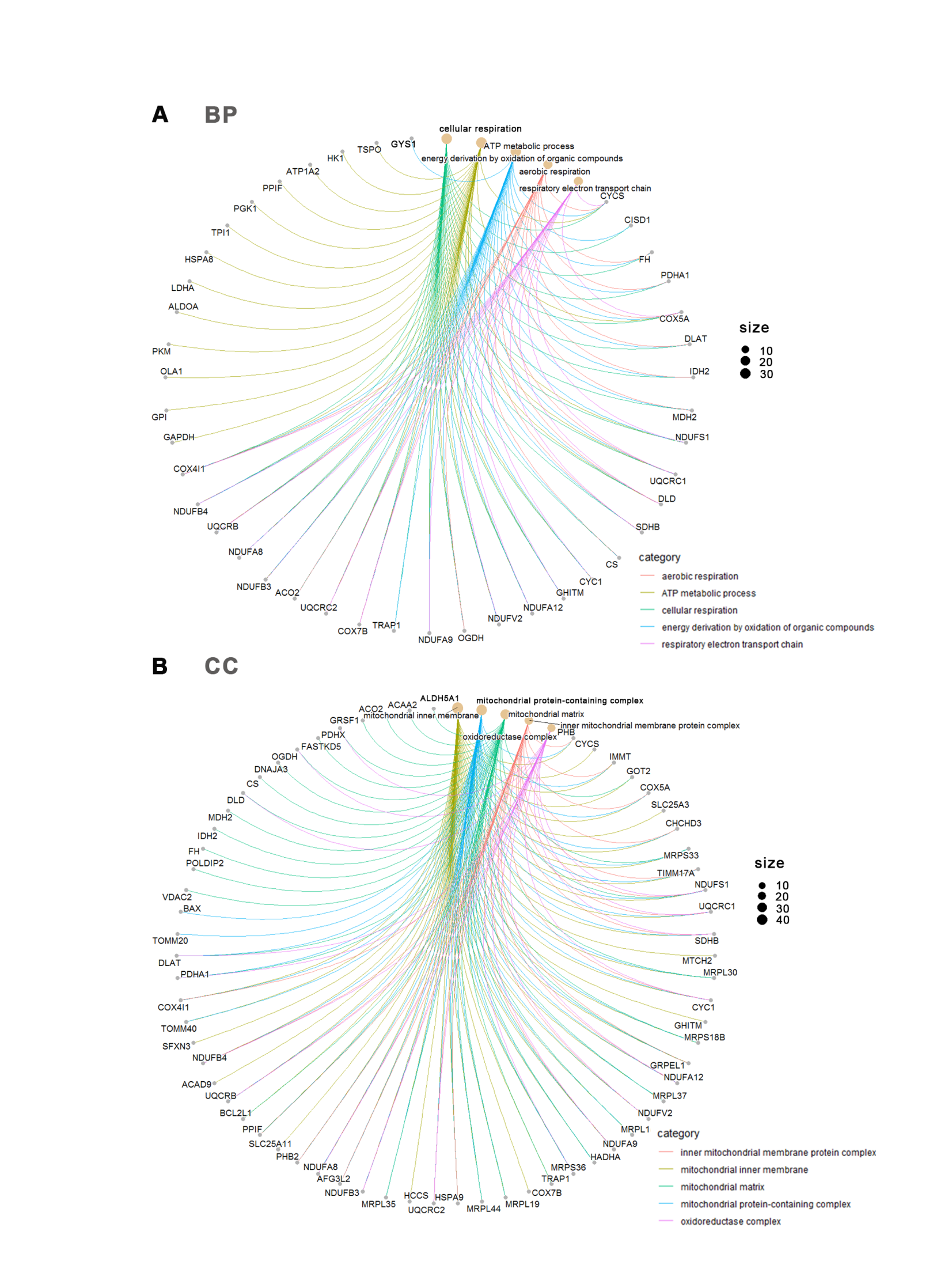


**Supplementary Figure 4.** GO enrichment analysis of VDAC1 related genes in a biological process or cellular component. The cnetplots of biological process **(A)** and cellular component **(B)** GO analysis were obtained according to VDAC1-binding proteins and related genes.

# Supplementary Tables

**Supplementary Table 1.** Subgroup analysis on the correlation of VDAC1 expression and prognosis of breast cancer cases.

| **Factor** | **Subgroup** | **Sample size** | **OS** | | **RFS** | | **DMFS** | |
| --- | --- | --- | --- | --- | --- | --- | --- | --- |
|  |  |  | **HR** | ***P*** | **HR** | ***P*** | **HR** | ***P*** |
| **ER status** | ER positive | 3499 | 1.32(0.96-1.81) | 0.086 | 1.14(0.98-1.33) | 0.0866 | 1.19(0.91-1.57) | 0.1994 |
|  | ER negative | 2168 | 1.29(0.91-1.81) | 0.15 | 0.85(0.68-1.05) | 0.13 | 0.71(0.53-0.94) | **0.017** |
| **TP53 status** | mutated | 272 | 0.46(0.16-1.31) | 0.14 | 1.37(0.83-2.26) | 0.22 | 0.57(0.26-1.23) | 0.15 |
|  | Wild type | 388 | 2.54(1.36-4.74) | **0.0024** | 1.39(0.83-2.34) | 0.21 | 2.41(1.15-5.02) | **0.016** |
| **PR status** | PR positive | 1559 | 2.38(0.9-6.28) | 0.072 | 1.43(1.06-1.91) | **0.017** | 0.83(0.52-1.31) | 0.42 |
|  | PR negative | 1989 | 1.75(1.08-2.85) | **0.022** | 1.13(0.88-1.45) | 0.33 | 0.71(0.51-0.99) | **0.04** |
| **HER2 status** | HER2 positive | 1273 | 0.82(0.55-1.22) | 0.33 | 1.32(1.04-1.67) | **0.023** | 1.2(0.86-1.68) | 0.28 |
|  | HER2 negative | 6262 | 1.35(1.07-1.71) | **0.011** | 1.41(1.25-1.59) | **1.8e-8** | 0.85(0.7-1.04) | 0.11 |
| **Grade** | Grade 1 | 576 | 1.73(0.71-4.22) | 0.22 | 1.38(0.79-2.39) | 0.25 | 0.54(0.24-1.21) | 0.13 |
|  | Grade 2 | 1795 | 1.49(1-2.22) | **0.049** | 1.08(0.87-1.35) | 0.47 | 0.78(0.58-1.04) | 0.092 |
|  | Grade 3 | 2058 | 0.64(0.46-0.88) | **0.0054** | 1.37(1.12-1.67) | **0.0017** | 1.25(0.97-1.63) | 0.09 |
| **Intrinsic subtype** | Basal | 1494 | 0.75(0.5-1.13) | 0.17 | 1.54(1.23-1.93) | **0.0002** | 0.71(0.5-1.01) | 0.052 |
|  | Luminal A | 3511 | 1.49(1.06-2.11) | **0.021** | 1.39(1.17-1.64) | **0.0002** | 1.21(0.93-1.58) | 0.15 |
|  | Luminal B | 2015 | 1.26(0.89-1.79) | 0.19 | 1.28(1.08-1.53) | **0.0055** | 0.82(0.6-1.11) | 0.19 |
|  | HER2+ | 515 | 0.63(0.35-1.12) | 0.11 | 1.77(1.24-2.54) | **0.0016** | 2.02(1.19-3.42) | **0.0077** |
| **Lymph node status** | Lymph node positive | 2153 | 0.5(0.36-0.71) | **4.9e-5** | 0.86(0.71-1.04) | 0.11 | 0.77(0.58-1.02) | 0.071 |
|  | Lymph node-negative | 2829 | 1.86(1.3-2.66) | **0.0006** | 1.14(0.96-1.35) | 0.12 | 1.32(1.03-1.7) | **0.03** |
| **Pietenpol subtype** | Basal-like 1 | 418 | 0.48(1.22-1.05) | 0.061 | 1.8(1.14-2.83) | **0.01** | 0.74(0.39-1.37) | 0.33 |
|  | Basal-like 2 | 165 | 5.26(1.19-23.15) | **0.014** | 3.84(1.93-7.66) | **4.0e-5** | 3.36(1.35-8.34) | **0.0056** |
|  | immunomodulatory | 462 | 0.52(0.22-1.24) | 0.13 | 0.54(0.33-0.87) | **0.011** | 0.38(0.22-0.67) | **0.0005** |
|  | Mesenchymal | 382 | 1.52(0.76-3.05) | 0.23 | 1.37(0.92-2.06) | 0.12 | 0.75(0.39-1.46) | 0.4 |
|  | Mesenchymal stem-like | 201 | 6.2(2.15-17.89) | **0.00014** | 2.09(0.99-4.42) | **0.047** | 5.35(1.81-15.81) | **0.00072** |
|  | Luminal androgen receptor | 413 | 0.58(0.32-1.07) | 0.08 | 1.47(0.98-2.2) | 0.064 | 0.45(0.25-0.81) | **0.0057** |

HR, hazard ratio; OS, overall survival; RFS, relapse-free survival; DMFS, distant metastasis-free survival;

ER, Estrogen receptor; PR, progesterone receptor; HER2, human epidermal growth factor receptor-2;

TP53, Tumor Protein P53; NA, not available data; *P* value less than 0.05 is shown bold.

**Supplementary Table 2.** Subgroup analysis on the correlation of VDAC1 expression and prognosis of lung cancer cases**.**

| **Factor** | **Subgroup** | **Sample size** | **OS** | | **FP** | | **PPS** | |
| --- | --- | --- | --- | --- | --- | --- | --- | --- |
|  |  |  | **HR** | ***P*** | **HR** | **HR** | ***P*** | **HR** |
| **Histology** | adenocarcinoma | 866 | 1.75(1.38-2.21) | **2.7e-6** | 1.53(1.12-2.1) | **0.0079** | 1.78(1.11-2.86) | **0.016** |
|  | squamous cell carcinoma | 675 | 0.88(0.69-1.12) | 0.29 | 0.67(0.4-1.13) | 0.13 | 0.42(0.12-1.49) | 0.16 |
| **gender** | female | 817 | 1.63(1.29-2.06) | **4.5e-5** | 0.8(0.59-1.07) | 0.14 | 1.52(1-2.32) | **0.048** |
|  | male | 1387 | 1.18(1-1.39) | **0.044** | 1.25(0.97-1.63) | 0.089 | 1.9(1.32-2.75) | **0.0005** |
| **smoking history** | exclude those never smoked | 970 | 1.47(1.19-1.83) | **0.0004** | 0.78(0.61-1.01) | 0.0561 | 1.76(1.28-2.41) | **0.0004** |
|  | only those never smoked | 247 | 1.72(0.97-3.06) | 0.0607 | 0.62(0.38-1.01) | 0.054 | 1.49(0.75-2.94) | 0.2495 |
| **stage** | stage I | 652 | 2.23(1.7-2.92) | **3.2e-9** | 1.59(1.02-2.46) | **0.0372** | 2.15(1.18-3.93) | **0.0107** |
|  | stage II | 320 | 0.55(0.35-0.84) | **0.0056** | 0.61(0.3-1.02) | 0.058 | 2.11(1.1-4.07) | **0.0225** |
|  | stage III | 70 | 1.66(0.89-3.07) | 0.11 | NA | NA | NA | NA |
| **grade** | grade I | 202 | 1.64(1.12-2.42) | **0.0105** | 1.66(0.95-2.92) | 0.072 | 1.97(1.19-3.24) | **0.0069** |
|  | grade II | 310 | 1.82(1.32-2.51) | **0.0002** | 2.17(1.42-3.31) | **0.0002** | 1.25(0.74-2.11) | 0.4033 |
|  | grade III | 77 | 2.18(1.1-4.32) | **0.0225** | 0.38(0.17-0.86) | **0.016** | 0.46(0.16-1.31) | 0.1353 |
| **AJCC stage t** | t1 | 475 | 1.28(0.95-1.73) | 0.1042 | 0.61(0.33-1.13) | 0.1129 | 0.76(0.4-1.42) | 0.3868 |
|  | t2 | 686 | 1.52(1.19-1.94) | **0.0008** | 0.67(0.49-0.9) | **0.0084** | 1.72(1.2-2.45) | **0.0027** |
|  | t3 | 99 | 1.31(0.79-2.16) | 0.2885 | 0.69(0.25-1.9) | 0.4702 | NA | NA |
|  | t4 | 48 | 1.7(0.89-3.23) | 0.1017 | NA | NA | NA | NA |
| **AJCC stage n** | n0 | 863 | 1.51(1.18-1.93) | **0.0011** | 0.64(0.43-0.97) | **0.0352** | 2.08(1.38-3.12) | **0.0004** |
|  | n1 | 296 | 1.15(0.84-1.58) | 0.3823 | 0.52(0.32-0.85) | **0.0073** | 0.6(0.33-1.09) | 0.0915 |
|  | n2 | 113 | 1.65(1.07-2.55) | **0.0221** | 2.28(1.12-4.63) | **0.0198** | 4.82(2.06-11.3) | **7.7e-5** |
| **AJCC stage m** | m0 | 818 | 0.9(0.73-1.11) | 0.3311 | 1.29(0.77-2.14) | 0.33 | 0.72(0.38-1.35) | 0.3019 |
| **surgery** | only surgical margins negative | 730 | 1.63(1.28-2.07) | **6.0e-5** | 1.37(1.05-1.78) | **0.0219** | 1.27(0.93-1.72) | 0.1267 |
| **radiotherapy** | no | 276 | 2.03(1.42-2.9) | **7.1e-5** | 1.84(1.26-2.68) | **0.0014** | 1.68(1.04-2.72) | **0.0331** |
|  | yes | 73 | 2.12(1.07-4.23) | **0.0282** | 0.68(0.4-1.16) | 0.152 | 2.87(1.28-6.46) | **0.0076** |
| **chemotherapy** | no | 317 | 2.21(1.58-3.08) | **2.0e-5** | 2.08(1.41-3.07) | **0.0002** | 2.01(1.25-3.23) | **0.0033** |
|  | yes | 178 | 0.74(0.48-1.13) | 0.1617 | 1.7(0.98-2.94) | 0.0546 | 0.67(0.4-1.15) | 0.1432 |

HR, hazard ratio; AJCC， American Joint Committee on Cancer; OS, overall survival; FP, first progression; PPS, post-progression survival; NA, not available data; P value less than 0.05 is shown in bold.

**Supplementary Table 3.** Subgroup analysis on the correlation of VDAC1 expression and prognosis of ovarian cancer cases.

| **Factor** | **Subgroup** | **Sample size** | **OS** | | **PFS** | | **PPS** | |
| --- | --- | --- | --- | --- | --- | --- | --- | --- |
|  |  |  | **HR** | ***p*** | **HR** | ***p*** | **HR** | ***p*** |
| **Histology** | Endometrioid | 62 | 0.56(0.09-3.35) | 0.52 | 0.61(0.23-1.63) | 0.32 | NA | NA |
|  | Serous | 1232 | 1.1(0.93-1.3) | 0.26 | 1.16(0.99-1.36) | 0.068 | 1.22(1-1.48) | **0.046** |
| **Stage** | Stage 1 | 107 | 2.05(0.61-0.68) | 0.24 | 1.9(0.65-5.54) | 0.23 | NA | NA |
|  | Stage 2 | 72 | 0.26(0.07-0.96) | **0.03** | 0.49(0.23-1.05) | 0.062 | 0.16(0.04-0.67) | **0.0049** |
|  | Stage 3 | 1079 | 1.21(1.02-1.44) | **0.028** | 1.27(1.07-1.5) | **0.0062** | 1.29(1.04-1.59) | **0.018** |
|  | Stage 4 | 189 | 1.25(0.83-1.88) | 0.29 | 1.68(1.07-2.65) | **0.024** | 1.53(0.95-2.45) | 0.076 |
| **Grade** | Grade 1 | 56 | 0.54(0.21-1.41) | 0.2 | 0.41(0.13-1.26) | 0.11 | NA | NA |
|  | Grade 2 | 325 | 0.77(0.57-1.05) | 0.095 | 1.24(0.92-1.67) | 0.16 | 0.78(0.52-1.15) | 0.21 |
|  | Grade 3 | 1024 | 1.07(0.9-1.26) | 0.45 | 1.14(0.94-1.37) | 0.18 | 1.24(0.99-1.56) | 0.064 |
|  | Grade 4 | 21 | 0.56(0.2-1.61) | 0.28 | NA | NA | NA | NA |
| **TP53 mutation** | Mutated | 516 | 1.41(1.1-1.79) | **0.0057** | 1.62(1.27-2.05) | **7.5e-05** | 1.55(1.15-2.09) | **0.0034** |
|  | Wild type | 102 | 0.61(0.31-1.17) | 0.13 | 1.56(0.91-2.68) | 0.1 | 0.62(0.32-1.22) | 0.17 |
| **Debulk** | optimal | 802 | 0.77(0.62-0.96) | **0.022** | 1.24(1-1.54) | **0.047** | 0.76(0.59-0.99) | **0.041** |
|  | suboptimal | 536 | 1.13(0.92-1.38) | 0.26 | 1.55(1.23-1.95) | **2e-04** | 1.39(1.02-1.9) | **0.036** |
| **Chemotherapy** | Contains platin | 1438 | 1.09(0.94-1.26) | 0.26 | 1.33(1.17-1.53) | **2.9e-05** | 1.2(0.99-1.46) | 0.07 |
|  | Contains Taxol | 821 | 0.92(0.76-1.11) | 0.39 | 1.2(0.99-1.45) | 0.06 | 1.23(0.96-1.58) | 0.095 |
|  | Contains Taxol+platin | 804 | 0.89(0.73-1.08) | 0.23 | 1.2(0.99-1.45) | 0.067 | 1.22(0.95-1.57) | 0.11 |
|  | Contains Avastin | 50 | 1.5(0.57-3.96) | 0.41 | 0.68(0.32-1.41) | 0.29 | 1.5(0.56-4.01) | 0.41 |
|  | Contains Docetaxel | 108 | 1.42(0.82-2.47) | 0.21 | 1.37(0.82-2.3) | 0.23 | 1.91(1.02-3.58) | **0.038** |
|  | Contains Gemcitabine | 135 | 0.73(0.47-1.12) | 0.15 | 1.41(0.94-2.14) | 0.098 | 1.49(0.91-2.42) | 0.11 |
|  | Contains Paclitaxel | 248 | 1.27(0.79-2.04) | 0.32 | 1.26(0.87-1.83) | 0.22 | 1.37(0.81-2.33) | 0.24 |
|  | Contains Topotecan | 119 | 0.8(0.55-1.18) | 0.26 | 1.79(1.1-2.9) | **0.017** | 1.23(0.8-1.89) | 0.35 |

HR, hazard ratio; OS, overall survival; PFS, progression free survival; PPS, post-progression survival;

TP53, Tumor Protein P53; NA, not available data; P value less than 0.05 is shown bold.

**Supplementary Table 4.** Subgroup analysis on the correlation of VDAC1 expression and prognosis of gastric cancer cases.

| **Factor** | **Subgroup** | **Sample size** | **OS** | | **FP** | | **PPS** | |
| --- | --- | --- | --- | --- | --- | --- | --- | --- |
|  |  |  | **HR** | ***P*** | **HR** | ***P*** | **HR** | ***P*** |
| **Gender** | Female | 244 | 0.39(0.26-0.58) | **1.4e-6** | 0.42(0.28-0.64) | **2.4e-5** | 0.35(0.23-0.55) | **2.3e-6** |
|  | Male | 566 | 0.57(0.45-0.73) | **3.1e-6** | 0.58(0.45-0.75) | **1.9e-5** | 0.47(0.36-0.62) | **1.6e-8** |
| **Stage** | Stage 1 | 69 | 0.27(0.09-0.79) | **0.011** | 0.17(0.02-1.31) | 0.053 | 0.07(0.01-0.56) | **0.0012** |
|  | Stage 2 | 145 | 0.61(0.33-1.13) | 0.11 | 0.63(0.35-1.15) | 0.13 | 0.6(0.31-1.16) | 0.12 |
|  | Stage 3 | 319 | 0.47(0.34-0.67) | **1.6e-5** | 0.54(0.36-0.8) | **0.0016** | 0.36(0.23-0.56) | **2.7e-6** |
|  | Stage 4 | 152 | 0.5(0.33-0.76) | **0.00084** | 0.5(0.32-0.79) | **0.0025** | 0.47(0.29-0.74) | **0.001** |
| **Stage t** | t2 | 253 | 0.71(0.46-1.09) | 0.11 | 0.67(0.44-1.02) | 0.061 | 0.59(0.37-0.92) | **0.02** |
|  | t3 | 208 | 0.56(0.38-0.82) | **0.0023** | 0.68(0.47-0.97) | **0.035** | 0.42(0.28-0.63) | **1.6e-5** |
| **Stage n** | n0 | 76 | 0.34(0.1-1.18) | 0.074 | 0.38(0.11-1.29) | 0.11 | 0.26(0.07-0.97) | **0.031** |
|  | n1 | 232 | 0.42(0.27-0.64) | **4.7e-5** | 0.42(0.27-0.64) | **4.8e-5** | 0.4(0.25-0.63) | **4.3e-5** |
|  | n2 | 129 | 0.4(0.25-0.64) | **6e-5** | 0.51(0.33-0.79) | **0.0021** | 0.34(0.21-0.55) | **4.7e-6** |
|  | n3 | 76 | 0.43(0.23-0.78) | **0.0047** | 0.44(0.24-0.79) | **0.0053** | 0.5(0.27-0.91) | **0.02** |
| **Stage m** | m0 | 459 | 0.58(0.44-0.77) | **0.00012** | 0.64(0.49-0.85) | **0.0014** | 0.43(0.32-0.58) | **1.6e-8** |
|  | m1 | 58 | 0.38(0.2-0.71) | **0.0016** | 0.45(0.22-0.93) | **0.026** | 0.48(0.2-1.14) | 0.09 |
| **HER2** | negative | 641 | 0.53(0.41-0.67) | **9.2e-8** | 0.53(0.4-0.69) | **2.5e-6** | 0.44(0.33-0.58) | **7.3e-9** |
|  | positive | 424 | 0.63(0.46-0.85) | **0.0026** | 0.57(0.39-0.82) | **0.0025** | 0.48(0.33-0.7) | **9.7e-5** |
| **Lauren classification** | Instestinal | 336 | 0.39(0.28-0.54) | **3.1e-9** | 0.54(0.37-0.78) | **0.0011** | 0.37(0.25-0.56) | **9.2e-7** |
|  | Diffuse | 248 | 0.57(0.4-0.81) | **0.0018** | 0.54(0.38-0.77) | **0.00055** | 0.55(0.37-0.81) | **0.0022** |
|  | Mixed | 33 | 0.44(0.12-1.58) | 0.2 | 2.31(0.8-6.62) | 0.11 | NA | NA |
| **Differentiation** | Poorly | 166 | 1.27(0.8-2.02) | 0.31 | 0.81(0.51-1.29) | 0.37 | 2.15(1.04-4.42) | **0.035** |
|  | Moderately | 67 | 0.47(0.24-0.93) | **0.027** | 0.53(0.28-1.01) | 0.051 | 1.65(0.64-4.26) | 0.29 |
|  | Well | 32 | 0.35(0.12-1.04) | **0.049** | NA | NA | NA | NA |
| **Treatment** | Surgery alone | 393 | 0.65(0.47-0.89) | **0.0069** | 0.71(0.53-0.96) | **0.027** | 0.51(0.37-0.69) | **1.2e-5** |
|  | 5-Fu based adjuvant | 157 | 1.25(0.88-1.78) | 0.21 | 0.84(0.56-1.26) | 0.4 | 1.34(0.95-1.91) | 0.098 |
| **Perforation** | No | 169 | 0.72(0.47-1.09) | 0.12 | 0.69(0.46-1.03) | 0.069 | 2.02(1.14-3.59) | **0.015** |

HR, hazard ratio; OS, overall survival; FP, first progression; PPS, post-progression survival;

HER2, human epidermal growth factor receptor-2; NA, not available data; P value less than 0.05 is shown in bold.

**Supplementary Table 5.** Subgroup analysis on the correlation of VDAC1 expression and prognosis of liver cancer cases.

| **Factor** | **Subgroup** | **Sample size** | **OS** | | **PFS** | | **RFS** | | **DSS** | |
| --- | --- | --- | --- | --- | --- | --- | --- | --- | --- | --- |
|  |  |  | **HR** | ***P*** | **HR** | ***P*** | **HR** | ***P*** | **HR** | ***P*** |
| **Stage** | Stage 1 | 171 | 2.09(1.14-3.84) | **0.015** | 1.42 (0.79-2.58) | 0.24 | 1.49(0.85-2.62) | 0.16 | 2.74(1.13-6.66) | **0.02** |
|  | Stage 2 | 86 | 0.26(0.08-0.88) | **0.021** | 1.75 (0.96-3.18) | 0.065 | 1.94(0.99-3.81) | **0.05** | 0.18(0.02-1.41) | 0.067 |
|  | Stage 3 | 85 | 2.37(1.28-4.37) | **0.0047** | 1.5(0.87-2.59) | 0.14 | 1.41(0.77-2.61) | 0.27 | 2.86(1.37-5.99) | **0.0037** |
| **Grade** | Grade 1 | 55 | 2.57(0.74-8.9) | **0.12** | 2.86(1.27-6.46) | **0.0085** | 2.31(0.86-6.18) | 0.086 | 5.16(0.66-40.4) | 0.081 |
|  | Grade 2 | 177 | 0.71(0.38-1.31) | 0.27 | 1.28(0.82-1.99) | 0.27 | 1.35(0.83-2.22) | 0.23 | 1.44(0.74-2.79) | 0.28 |
|  | Grade 3 | 122 | 1.82(1-3.31) | **0.048** | 0.68(0.37-1.26) | 0.22 | 0.58(0.33-1.02) | 0.057 | 1.54(0.71-3.32) | 0.27 |
| **AJCC_T** | T1 | 181 | 1.88(1.04-3.38) | **0.033** | 1.4(0.79-2.49) | 0.25 | 1.42(0.83-2.45) | 0.2 | 1.95(0.87-4.36) | 0.1 |
|  | T2 | 94 | 0.32(0.11-0.93) | **0.028** | 1.59(0.91-2.76) | 0.098 | 1.65(0.88-3.1) | 0.11 | 0.45(0.13-1.57) | 0.2 |
|  | T3 | 80 | 2.2(1.17-4.13) | **0.012** | 1.26(0.71-2.23) | 0.43 | 0.68(0.36-1.29) | 0.23 | 2.31(1.08-4.96) | **0.027** |
| **Gender** | Female | 121 | 1.48(0.85-2.58) | 0.16 | 0.77(0.45-1.32) | 0.35 | 0.73(0.4-1.35) | 0.32 | 1.41(0.69-2.88) | 0.34 |
|  | Male | 250 | 2.11(1.35-3.29) | **0.00078** | 1.55(1.08-2.23) | **0.017** | 1.43(0.95-2.14) | 0.081 | 2.24(1.26-3.98) | **0.0045** |
| **Vascular invasion** | None | 205 | 1.38(0.82-2.33) | 0.22 | 1.3(0.83-2.03) | 0.25 | 1.43(0.87-2.35) | 0.15 | 1.62(0.78-3.34) | 0.19 |
|  | micro | 93 | 1.65(0.76-3.6) | 0.2 | 1.31(0.72-2.37) | 0.37 | 1.44(0.76-2.71) | 0.26 | 1.98(0.66-5.89) | 0.21 |
| **Race** | White | 184 | 1.23(0.78-1.94) | 0.37 | 1.45(0.93-2.28) | 0.1 | 1.56(0.95-2.54) | 0.076 | 1.4(0.8-2.43) | 0.24 |
|  | Asian | 158 | 2.4(1.31-4.4) | **0.0036** | 1.6(1-2.57) | **0.05** | 1.48(0.89-2.46) | 0.12 | 2.68(1.16-6.21) | **0.017** |
| **Sorafenib treatment** | treated | 30 | 8.99(1.14-70.92) | **0.013** | 2.06(0.76-5.61) | 0.15 | 3.17(0.87-11.61) | 0.07 | 8.99(1.14-70.92) | **0.013** |
| **Alcohol consumption** | Yes | 117 | 2.69(1.37-5.29) | **0.0028** | 2.27(1.35-3.82) | **0.0016** | 2.09(1.14-3.81) | **0.014** | 3.06(1.43-6.58) | **0.0026** |
|  | none | 205 | 1.75(1.08-2.84) | **0.021** | 1.23(0.81-1.87) | 0.33 | 1.25(0.79-1.99) | 0.33 | 1.72(0.91-3.25) | 0.093 |
| **Hepatitis virus** | Yes | 153 | 1.91(1-3.64) | **0.047** | 1.53(0.96-2.42) | 0.07 | 1.56(0.95-2.57) | 0.077 | 1.95(0.86-4.45) | 0.1 |
|  | none | 169 | 2.26(1.4-3.65) | **6e-4** | 1.72(1.06-2.78) | **0.025** | 1.49 (0.86-2.57) | 0.15 | 2.15(1.38-4.56) | **0.0019** |

HR, hazard ratio; AJCC， American Joint Committee on Cancer; OS, overall survival; PFS, progress free survival;

RFS, relapse-free survival; DSS, disease-specific survival; NA, not available data; P value less than 0.05 is shown in bold.

**Supplementary Table 6.** Analysis of CPTAC-identified phosphorylation sites of VDAC1 via the PhosphoNET database.

| **site** | **sequence** | **experimentally confirmed^#^** | **hydrophobicity** | **p-site similarity score** | **maximum kinase specificity** | **sum kinase specificity score** | **conservation score** |
| --- | --- | --- | --- | --- | --- | --- | --- |
| **S102** | LKLTFDS**S**FSPNTGK | 15302935 | -0.653 | -56.8 | 447 | 18,129 | 31.8 |
| **S104** | LTFDSSF**S**PNTGKKN | 18707149 | -1.140 | -57.2 | 443 | 18,390 | 31.8 |

#The PMID (PubMed Unique Identifier) information of the publication was provided; NA, not available.
